# Identification of a Short Cell-Penetrating Peptide from Bovine Lactoferricin for Intracellular Delivery of DNA in Human A549 Cells

**DOI:** 10.1371/journal.pone.0150439

**Published:** 2016-03-04

**Authors:** Betty R. Liu, Yue-Wern Huang, Robert S. Aronstam, Han-Jung Lee

**Affiliations:** 1 Department of Natural Resources and Environmental Studies, National Dong Hwa University, Hualien, 974, Taiwan; 2 Department of Biological Sciences, Missouri University of Science and Technology, Rolla, Missouri, 65409–1120, United States of America; 3 College of Science and Technology, Bloomsburg University of Pennsylvania, Bloomsburg, Pennsylvania, 17815–1301, United States of America; University of Helsinki, FINLAND

## Abstract

Cell-penetrating peptides (CPPs) have been shown to deliver cargos, including protein, DNA, RNA, and nanomaterials, in fully active forms into live cells. Most of the CPP sequences in use today are based on non-native proteins that may be immunogenic. Here we demonstrate that the L5a CPP (RRWQW) from bovine lactoferricin (LFcin), stably and noncovalently complexed with plasmid DNA and prepared at an optimal nitrogen/phosphate ratio of 12, is able to efficiently enter into human lung cancer A549 cells. The L5a CPP delivered a plasmid containing the enhanced green fluorescent protein (*EGFP*) coding sequence that was subsequently expressed in cells, as revealed by real-time PCR and fluorescent microscopy at the mRNA and protein levels, respectively. Treatment with calcium chloride increased the level of gene expression, without affecting CPP-mediated transfection efficiency. Zeta-potential analysis revealed that positively electrostatic interactions of CPP/DNA complexes correlated with CPP-mediated transport. The L5a and L5a/DNA complexes were not cytotoxic. This biomimetic LFcin L5a represents one of the shortest effective CPPs and could be a promising lead peptide with less immunogenic for DNA delivery in gene therapy.

## Introduction

Small polar molecules, such as ions, amino acids, and monosaccharides, are able to enter cells through specific carriers or channels in the plasma membrane. The intrinsic nature of cellular membranes precludes influx of macromolecules, such as DNAs, RNAs, and proteins [1]. Several strategies have been developed to deliver exogenous macromolecules across cellular membranes. These include mechanical and electrical transfection techniques, such as microinjection, bioballistics, hydrodynamic force, ultrasonic nebulization, and electroporation, and chemical/biochemical methods, such as calcium phosphate coprecipitation, and membrane permeation catalyzed by artificial lipids, peptides/proteins, dendrimers, and virus-based vectors [2]. Some of these methods are suitable for *in vitro* or *in vivo* use, while others are suitable for both. For safety reasons, nonviral delivery methods, such as peptide- and lipid-based systems, have received more attention over the past twenty years than viral methods. Advantages of nonviral systems include ease and flexibility of assembly, minimal toxicity, and low levels of immunogenicity and insertional mutagenesis.

Cell-penetrating peptides (CPPs) that can deliver therapeutic and diagnostic molecules into cells in a nontoxic manner have recently received considerable attention as a promising nonviral tool for the delivery of drugs and diagnostic agents [1,2]. The first CPP discovered, transactivator of transcription (Tat)-protein transduction domain (PTD), consists of eleven amino acids (YGRKKRRQRRR) of the HIV-1 Tat. Tat-PTD is rich in basic amino acids, and is required for Tat translocation through the plasma membrane [3]. Subsequently, a variety of amphipathic, hydrophobic, and cationic peptides with less than thirty amino acids in length were identified and found to be able to deliver a wide range of biological cargos into cells [4]. Approximately 1,700 CPP sequences have been identified and collected in database CPPsite 2.0 [5] (http://crdd.osdd.net/raghava/cppsite/). The CPPpred (http://bioware.ucd.ie/~compass/biowareweb/Server_pages/cpppred.php) and CellPPD (http://crdd.osdd.net/raghava/cellppd/submission.php) websites provided tools that predict CPP effectiveness [6,7]. A quantitative structure-activity relationship (QSAR) model was recently developed that predicts the physiochemical properties of amphipathic CPPs [8]. However, the mechanisms by which CPPs and CPP/cargo complexes traverse cell membranes remain incompletely understood.

Lactoferrin (LF), an 80-kDa glycoprotein with iron-binding ability, is present in most biological fluids of mammals, including milk, saliva, tears, and mucous secretions [9]. Hydrolysates prepared from cleavage of LF with pepsin have strong antibacterial activity [10]. The antimicrobial peptide lactoferricin (LFcin) is located in the N-terminal region of LF [11]. The primary structure of bovine LFcin consists of a loop of 25 amino acids (residues 17–41 of the parent LF sequence [12]) formed by a disulfide bond between cysteine residues 19 and 36 [11]. Many LFcin derivatives possess antiviral [13,14], antifungal [15,16], antimicrobial [17–21], antitumoral [22], antiprotozoal [23], anticancer [9,24], and antihypertensive [25] activities (for a review [26]). Recently, the antimicrobial core of bovine LFcin has been narrowed down to only six amino acids (RRWQWR) [24,25].

A 22-amino acid loop form LFcin was the first CPP isolated from the N-terminal domain of human LF [27], which corresponds to amino acid residues 19–40 in bovine LF [28]. This loop structure formed by a disulfide bond between cysteine residues 20 and 37 is strictly conformation-dependent for efficient uptake into cells [27]. Binding of human LFcin to negatively charged heparin sulfates at the cell surface was the driving force for cellular uptake of arginine-rich CPPs [29]. Subsequently, the bLFcin_6_ sequence (RRWQWR) was identified from bovine as a new CPP that can effectively deliver small interfering RNA (siRNA) [30]. In contrast, the CPP_5_ (RWQWR), one of the shortest CPPs described [31], has less internalization activity [30]. Recently, a systematical study using human proteomic databases screened amino acid sequences of peptides or protein domains that reside or interact with cellular plasma membranes [32]. Fifty potential CPPs derived from 46 proteins were identified that could deliver siRNA across plasma membranes. Among them, three human CPPs derived from surfactant B, orexin, and LFcin were studied in further detail. It shall be noted that their published sequences of 25-amino acid LFcin and 12-amino acid LFcin (short) [32] are bovine sequences, not human sources.

Antimicrobial peptides play an important role in membrane destroying, alternation, or permeation, and some of them may have antibiotic activity [33]. Alternatively, other membrane interacting peptides that do not compromise membrane integrity are very important in modulating the structure and dynamics of the lipid bilayer, and thereby cell membrane function. It has long been appreciated that antimicrobial peptides and CPPs possess similar functional characteristics [33,34]. Thus, we suspected that bovine LFcin derived peptides with antimicrobial activity in prokaryotes could act as CPPs in eukaryotic cells. In the present study, a novel penta-peptide (L5a) from bovine LFcin was examined. This nontoxic L5a peptide was found to noncovalently deliver DNA into human cells.

## Materials and Methods

### Cell culture

Human bronchoalveolar carcinoma A549 cells (American Type Culture Collection, Manassas, VA, USA; CCL-185) were cultured in Roswell Park Memorial Institute (RPMI) 1640 medium (Gibco, Invitrogen, Carlsbad, CA, USA) supplemented with 10% (v/v) bovine serum (Gibco), as previously described [35]. In experiments exposing cells to CPPs and CPP/DNA complexes, cells were washed with phosphate buffered saline (PBS) three times, and the culture medium was switched to serum-free Opti-MEM I Reduced Serum Medium (Life Technologies, Thermo Fisher Scientific Inc., Waltham MA, USA).

### Preparation of peptide and plasmid DNA

The CPPs fluorescein isothiocyanate (FITC) N-labeled histidine-rich nona-arginine (CHHHHHRRRRRRRRRHHHHHC; denoted as HR9-FITC) [36], short LFcin (CRRWQWRMKKLGC; LFcin-FITC) [32], L12-FITC (FKCRRWQWRMKK), L11-FITC (KCRRWQWRMKK), L9-FITC (RRWQWRMKK), and L6-FITC (RRWQWR) were purchased from Genomics (Taipei, Taiwan). Other CPPs, including L6, L5a (RRWQW), L5a-FITC, L5b-FITC (RWQWR), and L4-FITC (RWQW), and nonCPP bradykinin-FITC (RPPGFSPFR) [37], were purchased from GMbiolab Co. (Taichung, Taiwan). Various amounts of CPPs were mixed with plasmid DNA and incubated at 37°C for designated periods of time. A molecular nitrogen (NH_3_^+^)/phosphate (PO_4_^−^) (N/P) ratio [38] between peptide and plasmid DNA of 12:1 promoted maximal stable CPP/DNA complex formation, as revealed by gel retardation assays, and this ratio was used in subsequent experiments.

The pEGFP-N1 plasmid contains the enhanced green fluorescent protein (*EGFP*) coding sequence under the control of the cytomegalovirus (CMV) promoter (Clontech, Mountain View, CA, USA). The pCAGGS-tdTomato plasmid consists of the tandem dimeric *Tomato* coding sequence under the control of the CMV early enhancer/chicken beta-actin (CAG) promoter [39]. The *EGFP* coding sequence was removed by the digestion of the pEGFP-N1 plasmid with both *Bam HI* and *Not I* restriction enzymes to generate the pN1 backbone plasmid.

### Noncovalent protein transduction

In dosage-dependent protein transduction experiments, A549 cells were treated with 0, 1, 5, 10 or 30 μM of FITC-labeled LFcin derivatives at 37°C for 1 h. In time-course studies of transfection, cells were treated with FITC-labeled LFcins at 37°C for 0, 5, 10, 20, 30 or 60 min. For cellular internalization analysis, cells were treated with 10 μM of FITC-labeled LFcins at 37°C for 1 h. Following protein transduction, the cells were washed with PBS and incubated at 37°C for an additional 48 h.

To determine nuclear localization, cells were treated with CPPs or CPP/DNA complexes at 37°C for 1 h, followed by fixation with 3.7% formaldehyde, as previously described [35]. Cells were stained with the fluorescent nuclear-specific tracker Hoechst 33342, according to the manufacturer's instructions (Invitrogen).

For the functional assay, cells were treated with CPP (14.4 μg L6 or 16.0 μg L5a) alone, 1.6 μg reporter gene plasmid alone or CPP/DNA complexes prepared at an N/P ratio of 12 at 37°C for 1 h in the absence or presence of 113 mM of CaCl_2_, as previously described [38]. Cells were treated with Lipofectamine 2000 (Life Technologies) premixed with DNA according to the manufacturer's instructions as a positive control. Following treatment, cells were washed with PBS and incubated at 37°C for an additional 48 h.

### Flow cytometric analysis

Human A549 cells were seeded onto 24-well plates. Cells in the control and the experimental groups were harvested and analyzed using the Cell Lab Quanta SC MPL flow cytometer (Beckman Coulter, Fullerton, CA, USA) using the 525BP filter (excitation at 488 nm and emission at 525 nm wavelength) on the Fluorescent Channel 1 (FL1) for green fluorescent protein (GFP) detection. Results are expressed as the percentage of the total cell population displaying green fluorescence.

### Fluorescent microscopy

Fluorescent and bright-field images were recorded using an AE31 inverted Epi-fluorescence microscope (Motic, Causeway Bay, Hong Kong) with an IS1000 eyepiece (Tucsen, Fujian, China) or an Eclipse 80i Epi-fluorescence microscope (Nikon, Tokyo, Japan) with the NIS-elements documentation software (Nikon). For the AE31 fluorescent microscope, excitation filters were set at 480/30, 350/50, and 560/40 nm for GFP, blue (BFP), and red fluorescent protein (RFP) channels, respectively. Emission filters were set at 535/40, 460/50, and 630/60 nm for GFP, BFP, and RFP channels, respectively. For the Eclipse 80i Epi-fluorescent microscope, excitation filters were set at 450–490, 340–380, and 510–560 nm for GFP, BFP, and RFP channels, respectively. Emission filters were set at 520, 435–485, and 590 nm for GFP, BFP, and RFP channels, respectively. Bright-field images were used to observe cell morphology. Intensity of fluorescent images was quantified using the UN-SCAN-IT software (Silk Scientific, Orem, UT, USA).

### Gel retardation assay

Various amounts of CPPs were complexed with 2 μg of the pEGFP-N1 plasmid DNA and incubated at 37°C for 1 h. Complexes formed at N/P ratios ranging from 0 (DNA only) to 18 were analyzed by electrophoresis on a 0.5% SeaKem Gold agarose gel (Lonza Group, Basel, Switzerland) in 1 × TBE buffer (89 mM of Tris-borate and 2 mM of EDTA, pH 8.3) at 100 V for 30 min [35]. Gels were stained with the SYBR Safe DNA gel stain (Life Technologies), and images were captured using the ChemiDoc XRS+ gel imaging system (Bio-Rad, Hercules, CA, USA) with an excitation wavelength at 302 nm of trans-UV light and with an emission wavelength at 548–630 nm of the standard filter. Data were analyzed using the Quantity One 1-D analysis software 4.6.9 (Bio-Rad). The relevant percentage of a DNA band shift migrating in the gel was defined as a reciprocal mobility ratio between minimal N/P ratio (N/P = 0) setting as 0% and maximal N/P ratio (N/P = 18) setting as 100%.

### Real-time PCR

For transcription analysis, quantitative real-time PCR was used to assess the *EGFP* expression in cells. Cells were either untreated (negative control) or treated with DNA (4 μg) alone, CPP alone (36 μg L6 or 40 μg L5a), Lipofectamine 2000/DNA or CPP/DNA complexes prepared at an N/P ratio of 12 at 37°C for 48 h. Total RNA was isolated using the TRIzol reagent (Life Technologies, Ambion), and cDNA was then synthesized using the SuperScript VILO cDNA synthesis kit, according to the manufacturer's instructions (Life Technologies, Invitrogen). Real-time PCR was conducted using the Power SYBR Green PCR master mix (Life Technologies, Applied Biosystems) with a set of the specific *EGFP* primers EGFP-QF (5'-TCGTGACCACCCTGACCTAC-3') and EGFP-QR (5'-TGCGCTCCTGGACGTAGCCTTC-3') [40] using the iQ5 real-time PCR detection system (Bio-Rad). The quantification of *EGFP* expression was normalized with human *18S rRNA* expression amplified with a set of the specific primers h18S-QF (5'-CGGCTACCACATCCAAGGAA-3') and h18S-QR (5'-GCTGGAATTACCGCGGCT-3') [41] as internal standards.

### Zeta-potential measurement

CPP (10 μM) alone, DNA (2 μg pEGFP-N1 plasmid) alone or CPP/DNA complexes prepared at N/P ratios of 9 and 12 were dissolved in double deionized water (pH 7). Each solution was temperature-equilibrated at 25°C for at least 2 min in a capillary DTS1070 cuvette (Malvern Instruments, Worcestershire, UK). Zeta-potentials of samples were measured using a Zetasizer Nano ZS and analyzed with the Zetasizer software 6.30 (Malvern Instruments) [42].

### Cytotoxicity assay

To assess the cytotoxicity of CPPs, the pEGFP-N1 plasmid DNA and CPP/DNA complexes, cells were treated with 10 μM of CPP alone, 1.6 μg of DNA alone, Lipofectamine/DNA or CPP/DNA complexes prepared at an N/P ratio of 12 at 37°C for 24 h. A549 cells were treated with the serum-free medium and 100% DMSO for 24 h as negative and positive controls, respectively. The colorimetric 1-(4,5-dimethylthiazol-2-yl)-3,5-diphenylformazan (MTT) dye reduction assay was performed as previously described [35,43].

### Statistical analysis

Data are expressed as mean ± standard deviation (SD). Mean values and SDs were calculated from at least three independent experiments carried out in triplicates for each treatment group. Statistical comparisons were performed by ANOVA and the Student's *t*-test, using levels of statistical significance of *P* < 0.05 (*, α) or 0.01 (**, αα), as indicated.

## Results

### Characterization of cellular internalization of LFcin derivatives

**Fig 1:**
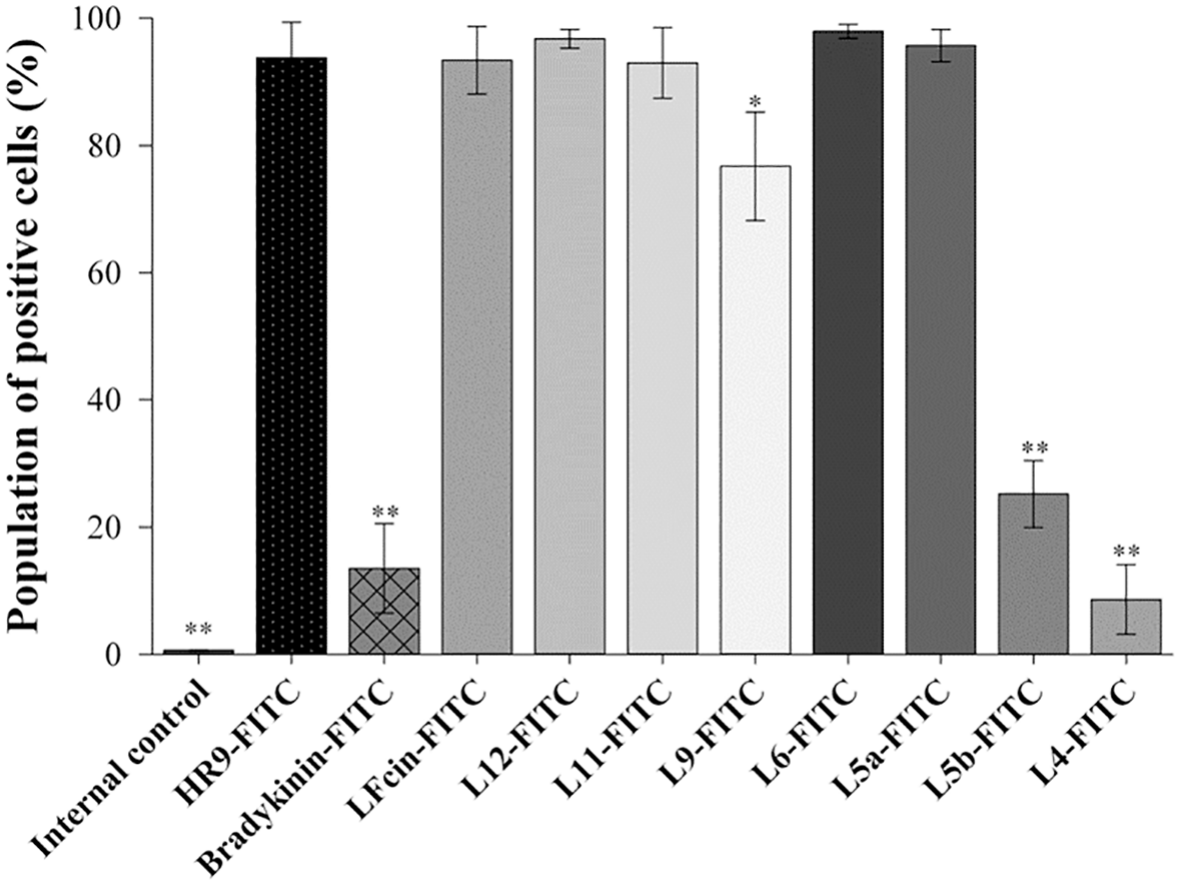
Cellular internalization of eight LFcin derivatives. Cells were treated with 10 μM FITC-labeled LFcins for 1 h. The fraction of cells internalizing the LFcin was determined by flow cytometry. Statistical comparisons were performed by ANOVA. Significant differences from the uptake of HR9-FITC at *P*<0.05 (*) and *P*<0.01 (**) are indicated. Data are presented as mean ± SD from seven independent experiments in each treatment group.

To determine cellular internalization of eight bovine LFcin derivatives of various lengths, human A549 cells were treated with FITC-labeled LFcins in different concentrations and then analyzed using a flow cytometer. Cellular internalization ability of L9-FITC peptide was dosage-dependent (S1 Fig). HR9-FITC, LFcin-FITC, L12-FITC, L11-FITC, L6-FITC, and L5a-FITC were internalized at low concentrations (< 1 μM), while nonCPP bradykinin-FITC (negative control), L5b-FITC, and L4-FITC displayed less internalization. To understand the rate of cellular internalization of LFcins, cells were treated with various peptides for different periods of time and then analyzed using a flow cytometer. HR9-FITC (positive control), L12-FITC, L11-FITC, L6-FITC, and L5a-FITC entered cells within 5 min (S2 Fig). The population of positive cells in LFcin-FITC, L12-FITC, L11-FITC, L6-FITC, and L5a-FITC treated groups was similar to that of the positive control group (Fig 1). However, the fraction of positive cells in L5b-FITC and L4-FITC treated groups was similar to that of bradykinin-FITC negative control group. It shall be noted that the internalization abilities of L5a-FITC and L5b-FITC, peptides of the same length but different sequence, was quite different. L5a is one the shortest CPPs identified thus far.

**Fig 2:**
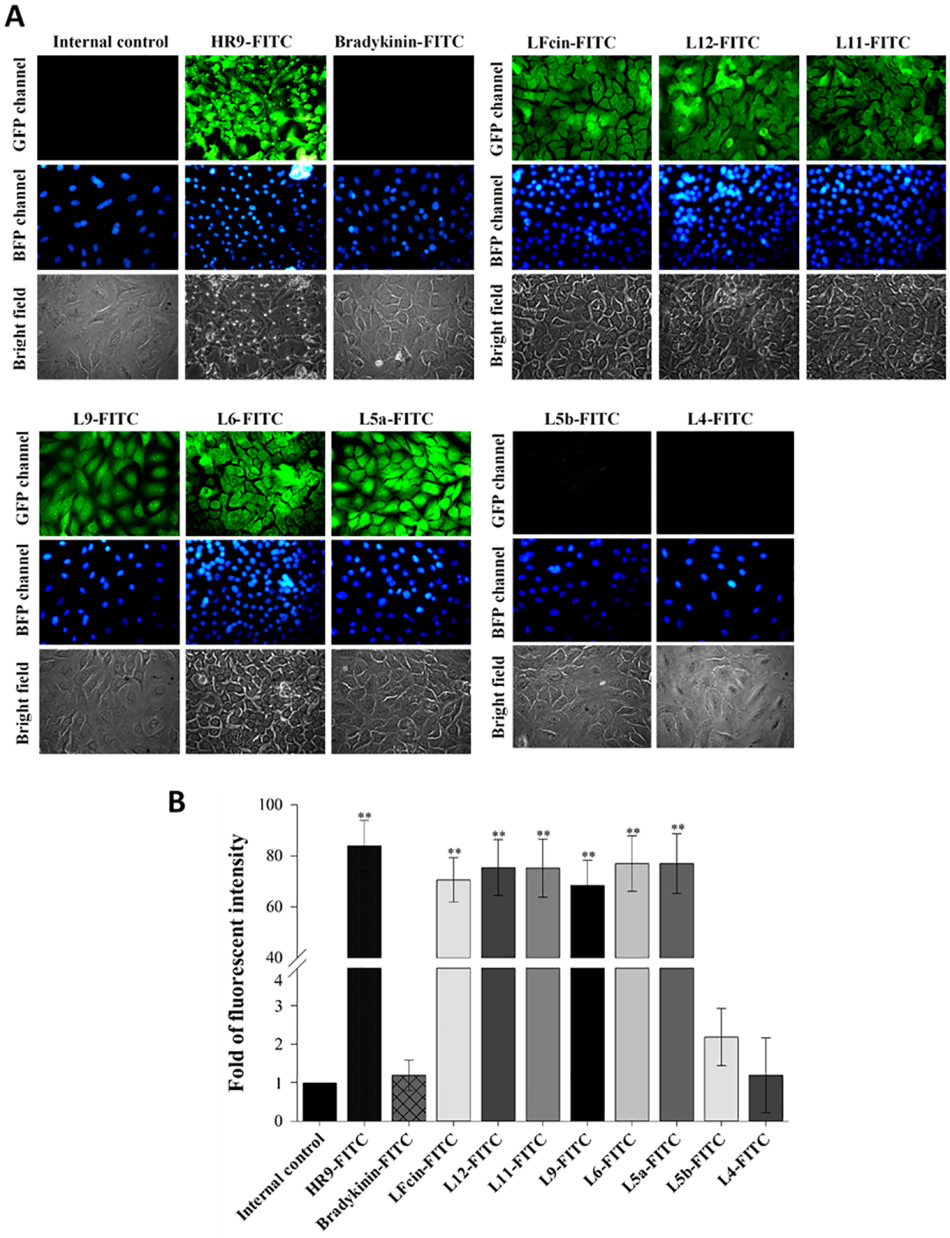
Fluorescent microscopy of cellular uptake of eight FITC-labeled LFcin derivatives into A549 cells. (A) Fluorescent images of cells treated with FITC-labeled LFcins. Cells were treated with 10 μM FITC-labeled LFcins for 1 h, and then stained with Hoechst 33342. Cells not exposed to LFcins served as internal controls. Cells were treated with HR9-FITC and bradykinin-FITC as positive and negative controls, respectively. GFP, BFP channels and bright fields revealed the distributions of FITC-labeled LFcins, nuclei and cell morphologies. All images are obtained using a Motic AE31 fluorescent microscope with a magnification of 400×. (B) Quantification of the uptake of FITC-labeled LFcins. The fluorescent intensity of cellular internalization was quantified using UN-SCAN-IT software. Each experiment group was compared with the internal control and statistical differences were calculated using the Student's *t*-test. Significant differences from HR9-FITC at *P*<0.01 (**) are indicated. Data are presented as mean ± SD from five independent experiments in each treatment group.

Fluorescent microscopy was used to analyze cellular internalization of LFcins of various lengths. Green fluorescence was detected in the cells treated with HR9-FITC, LFcin-FITC, L12-FITC, L11-FITC, L9-FITC, L6-FITC, and L5a-FITC (Fig 2A). In contrast, little green fluorescence was detected in cells treated with L5b-FITC, and almost no GFP channel fluorescence was seen in internal control cells or the cells treated with bradykinin-FITC or L4-FITC. Results from these fluorescent analyses (Fig 2B) were in agreement with the observations from the flow cytometry shown in Fig 1C. Consequently, L5a, the shortest high activity CPPs identified, was chosen as a shuttle candidate to deliver DNA cargos in subsequent experiments.

### CPP-mediated DNA delivery into human cells

**Fig 3:**
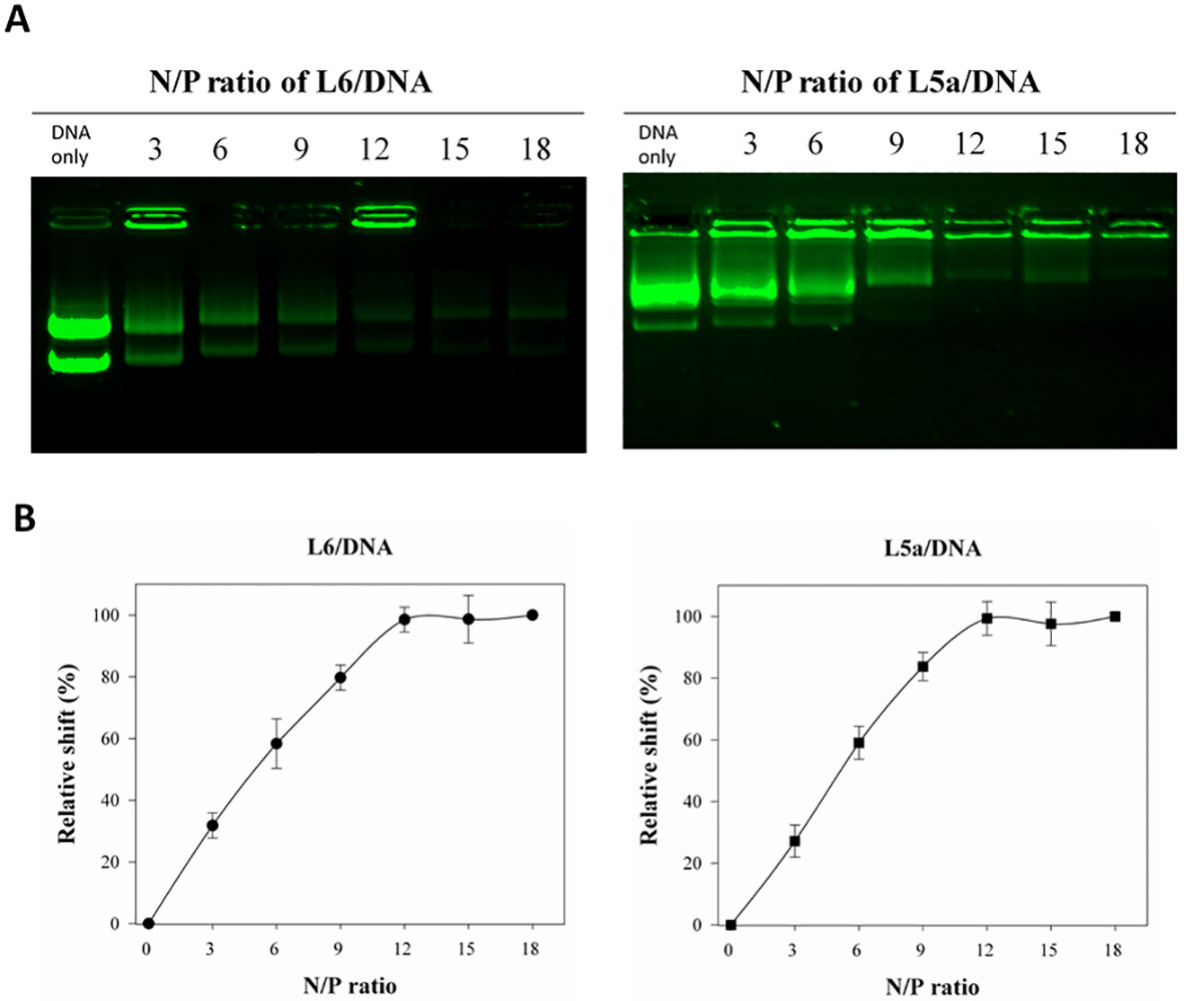
Noncovalent interactions between CPPs and plasmid DNA *in vitro*. (A) Gel retardation assay indicating the formation of CPP/DNA complexes. Various amounts of L6 or L5a were mixed with DNA at molecular N/P ratios of 0 (DNA only), 3, 6, 9, 12, 15, and 18. These complexes were analyzed by electrophoresis on a 0.5% agarose gel, followed by SYBR Safe staining. DNA images were captured using a ChemiDoc XRS+ Gel Imaging System (Bio-Rad). (B) The relative mobility of CPP/DNA complexes. The mobility of CPP/DNA complexes is indicated. Data are presented as mean ± SD from five independent experiments in each treatment group.

To determine whether L5a was able to interact with plasmid DNA to form noncovalent complexes *in vitro*, gel retardation assays were performed. Various amounts of L6 and L5a were complexed with DNA at N/P ratios of 0 (DNA only), 3, 6, 9, 12, 15, and 18. The gels were stained with *SBRY Safe* DNA gel stain (Fig 3A). Both L6 and L5a were able to noncovalently associate with DNA to form stable peptide/DNA complexes, and the degree of shift was related to the peptide content. Relative shift analysis revealed that ratio-dependent interactions of peptide/DNA complexes was maximal at N/P ratios above 12 (Fig 3B). This demonstrates that both L6 and L5a are able to interact with DNA to form stable complexes *in vitro*.

**Fig 4:**
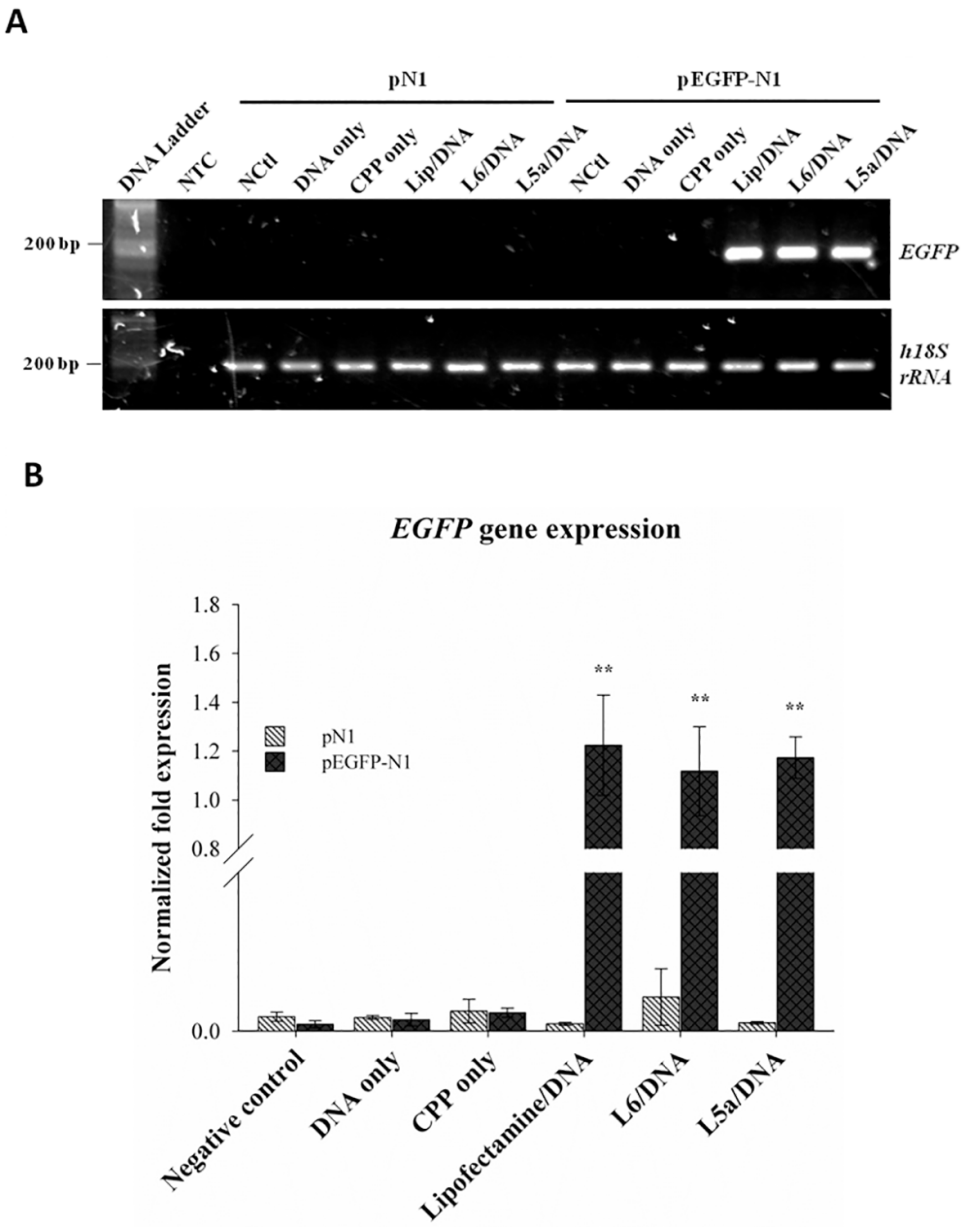
Real-time PCR analysis of cellular delivery of reporter gene plasmid mediated by L6 and L5a. (A) Final RT-PCR products of *EGFP* and *18S rRNA* genes from the cells after uptake of the peptide/DNA complexes. Cells were not treated (negative control, NCtl) or were treated with DNA alone, CPP alone, Lipofectamine 2000/DNA (Lip/DNA) or CPP/DNA complexes for 24 h. Real-time PCR for the detection of *EGFP* gene expression was conducted using a Bio-Rad iQ5 Real-Time PCR Detection System. Human*18S rRNA* gene expression was analyzed as an internal control. Negative control (NTC) represents real-time PCR signal in the absence of a DNA template. (B) Real-time PCR analysis of *EGFP* gene expression. *EGFP* gene expression recorded in panel A was normalized to *18S rRNA* expression. Statistical comparisons were performed by ANOVA. Data are presented as mean ± SD from three independent experiments in each treatment group. Significant differences from cells without any treatments (negative control) at *P*<0.01 (**) are indicated.

To study the transfection ability of L6 and L5a *in vivo*, L6/ and L5a/DNA complexes prepared at an N/P ratio of 12 were incubated with A549 cells, and the reporter gene expression was detected at the mRNA level by real-time PCR analysis. The *EGFP* gene located on the pEGFP-N1 plasmid was the reporter gene, while the empty pN1 vector served as a negative control. Cells treated with medium (negative control), DNA only, CPP only, Lipofectamine/DNA, L6/DNA, or L5a/DNA complexes were harvested after incubation for 48 h. The mRNAs prepared from treatment groups of Lipofectamine/pEGFP-N1, L6/pEGFP-N1, and L5a/pEGFP-N1 showed the cDNA products of 111 bp length (Fig 4A). However, no product could be detected in other treatment groups. The internal control of the housekeeping gene product *h18S* rRNA was detected as a cDNA product of 186 bp length in all treatment groups. Thus, the *EGFP* gene expression in treatment groups of Lipofectamine/pEGFP-N1, L6/pEGFP-N1, and L5a/pEGFP-N1 was significantly higher than that of the negative control, DNA, and CPP alone after normalization with *h18S* rRNA (Fig 4B). Moreover, real-time PCR failed to detect the expression of *EGFP* gene product in any treatment groups containing the pN1 plasmid. These results demonstrate that L6 and L5a are able to deliver plasmid DNA into cells, and that the DNA delivered by the CPPs was transcribed into mRNA.

**Fig 5:**
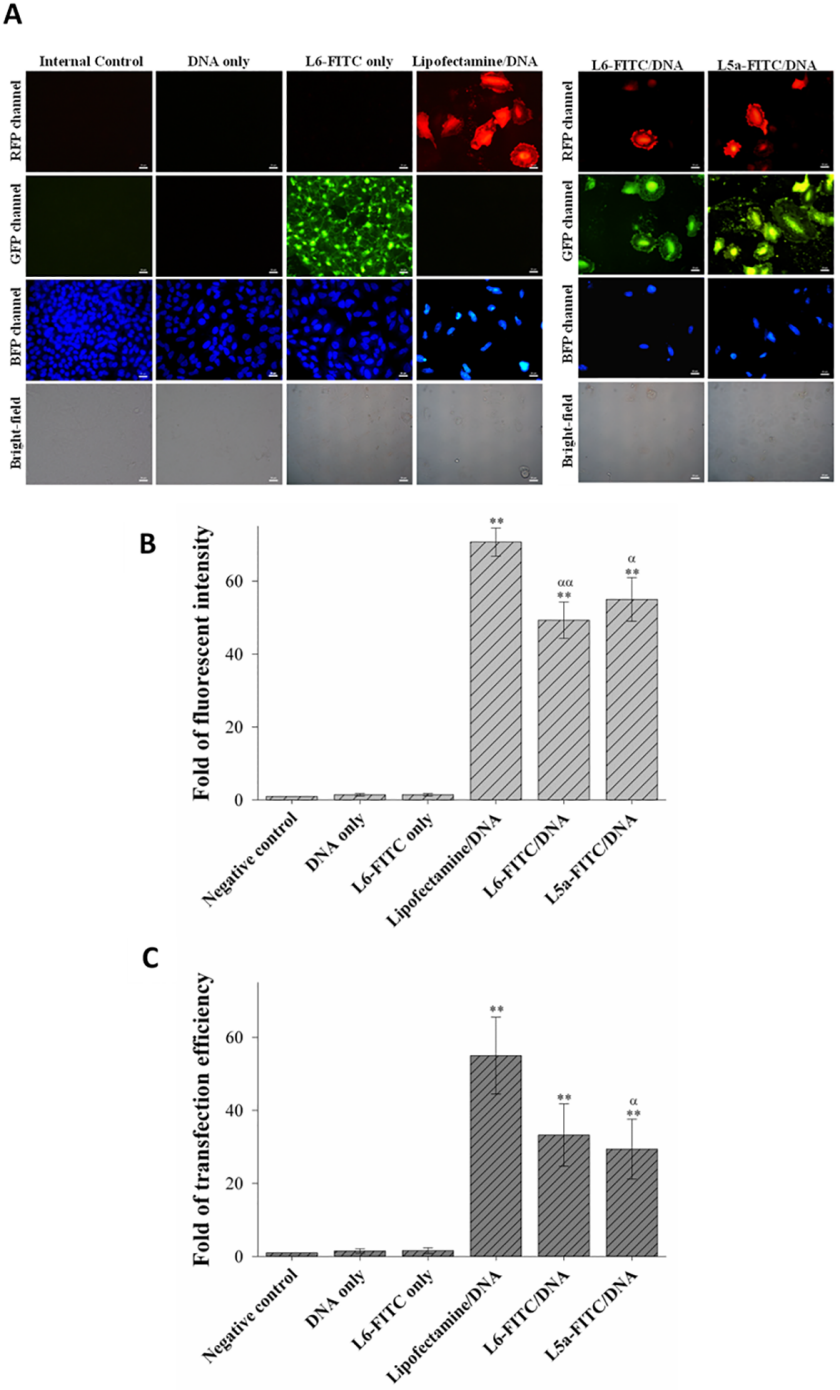
Functional assay of cellular delivery of reporter gene plasmid mediated by L6 and L5a. (A) Images of the FITC-labeled CPP-mediated delivery of the pCAGGS-tdTomato plasmid DNA into cells. L6-FITC or L5a-FITC was premixed with the pCAGGS-tdTomato plasmid at an N/P ratio of 12. These CPP/DNA complexes, DNA alone and CPP alone were incubated with A549 cells for 1 h. Untreated cells and cells treated with Lipofectamine 2000/DNA complexes served as negative and positive controls, respectively. Following the 1 h incubation, cells were washed with PBS and incubated at 37°C for an additional 48 h. BFP, GFP, and RFP channels displayed the distribution of nuclei stained by Hoechst33342, CPP-FITC, and tdTomato, respectively. Cell morphologies were observed in bright-field images. All images are obtained using a Nikon Eclipse 80i fluorescent microscope at a magnification of 1,000×. (B) Expression of reporter gene delivered by CPPs. Fluorescent intensity recorded in panel A was quantified using UN-SCAN-IT software, and analyzed by ANOVA. Data are presented as mean ± SD from three independent experiments in each treatment group. Experimental groups were compared with the negative control. Significant differences from the negative control at *P*<0.05 (α) and *P*<0.01 (**, αα) are indicated. (C) Transfection efficiency of CPP-mediated delivery of gene expression. Fold increases of transfection efficiency recorded in panel A were quantified using UN-SCAN-IT software. Statistical comparisons were performed by ANOVA. Data are presented as mean ± SD from three independent experiments in each treatment group. Significant differences from the negative control at *P*<0.05 (α) and *P*<0.01 (**) are indicated.

Fluorescent microscopy was used to observe reporter gene expression of the DNA delivered by CPPs at the protein level. A549 cells were treated with L6-FITC/ and L5a-FITC/pCAGGS-tdTomato plasmid DNA complexes prepared at an N/P ratio of 12. After a 48 h incubation, the cells were stained with Hoechst 33342 and observed with a fluorescent microscope. Cells treated with L6-FITC alone and Lipofectamine/pCAGGS-tdTomato complexes served as the positive controls (Fig 5A). Neither green nor red fluorescence was detected in the cells treated with PBS (internal control) or DNA alone. However, green fluorescence was observed in the cells treated with L6-FITC alone, and red fluorescence was detected in the Lipofectamine/DNA-treated cells. Both red and green fluorescence could be detected in the cells treated with L6-FITC/DNA and L5a-FITC/DNA complexes. In a quantitative analysis of fluorescent intensity, red fluorescence was higher in treatment groups of Lipofectamine/DNA, L6-FITC/DNA, and L5a-FITC complexes than that of the negative control, DNA, or CPP alone groups (Fig 5B). Maximal intensity of the reporter gene expression was observed in the cells treated with Lipofectamine/DNA complexes. However, the transfection efficiency in L6-FITC/DNA and Lipofectamine/DNA treatment groups was not different (Fig 5C). These results indicate that both L6 and L5a can efficiently deliver DNA into cells.

### Influence of calcium chloride on CPP-mediated DNA delivery into cells

**Fig 6:**
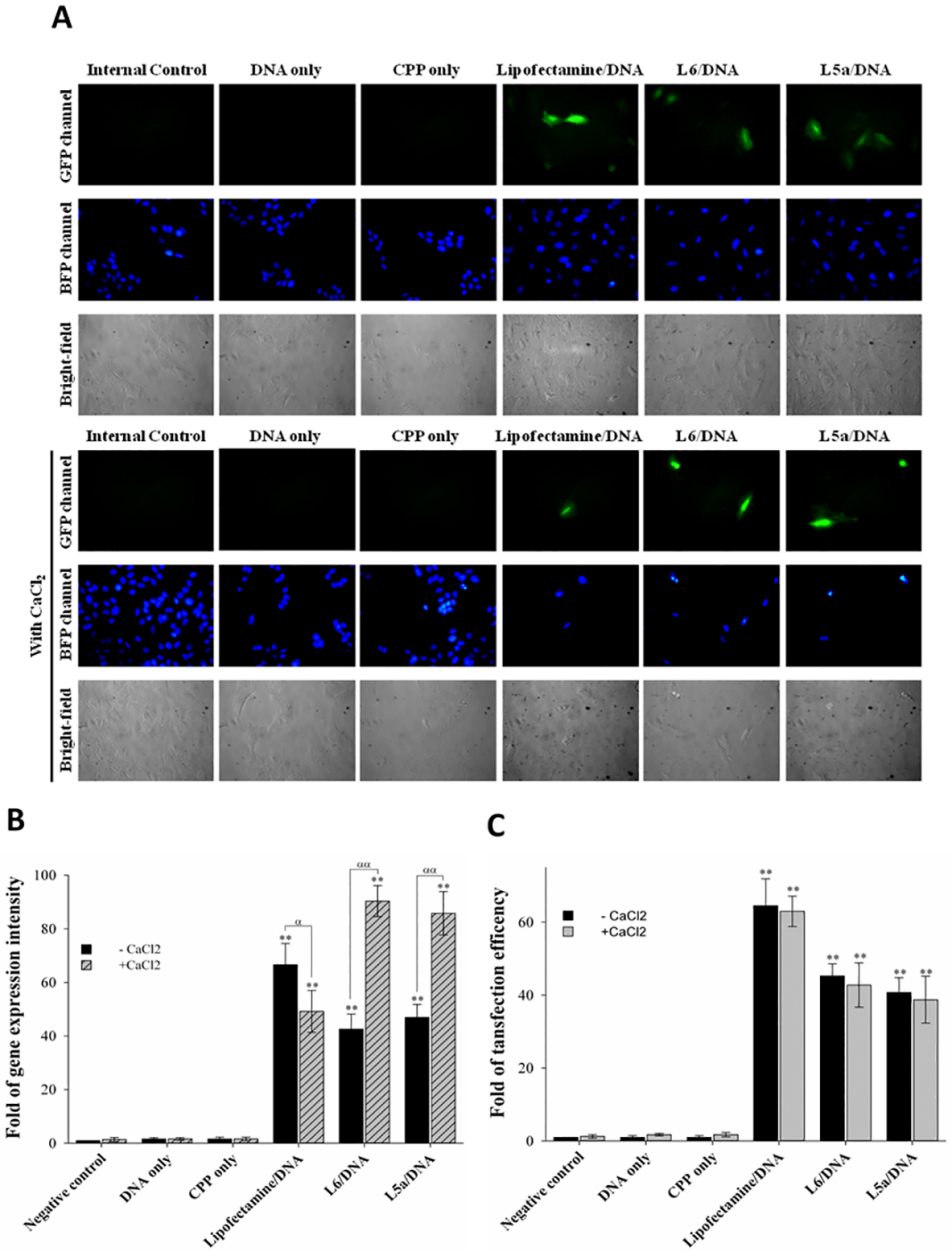
Influence of calcium chloride on CPP-mediated gene delivery. (A) Images of CPP-mediated delivery of the pEGFP-N1 plasmid DNA into cells in the absence or presence of calcium chloride. L6 or L5a was premixed with the pEGFP-N1 plasmid at an N/P ratio of 12. These CPP/DNA complexes, DNA alone, and CPP alone were incubated with cells for 1 h in the absence or presence of 113 mM CaCl_2_. Cells without any treatments and cells treated with Lipofectamine 2000/DNA complexes served as negative and positive controls, respectively. BFP and GFP channels revealed the distribution of nuclei stained by Hoechst33342 and EGFP, respectively. Cell morphologies were observed in bright-field images. All images are obtained using a Motic AE31 fluorescent microscope. (B) Fluorescent intensity of CPP-mediated delivery of gene expression. Fluorescent intensity recorded in panel A was quantified using UN-SCAN-IT software. Statistical comparisons were performed by ANOVA. Data are presented as mean ± SD from nine independent experiments in each treatment group. Experimental groups were compared with the negative control, and each group without CaCl_2_ treatment was compared with the group with CaCl_2_ treatment. Significant differences at *P*<0.05 (α) and *P*<0.01 (**, αα) are indicated. (C) Transfection efficiency of CPP-mediated delivery of gene expression. Folds of transfection efficiency recorded in panel A were quantified using UN-SCAN-IT software. Data are presented as mean ± SD from nine independent experiments in each treatment group. Significant differences at *P*<0.01 (**) are indicated.

To understand effects of calcium chloride on CPP-mediated DNA delivery, cells were treated with L6/pEGFP-N1and L5a/pEGFP-N1 complexes prepared at an N/P ratio of 12 in the absence or presence of calcium chloride, followed by staining with Hoechst 33342. Cells treated with Lipofectamine/DNA complexes served as the positive control. No signal was detected in the cells treated with PBS (internal control), pEGFP-N1 plasmid DNA, or CPP alone (Fig 6A). In contrast, green fluorescence was observed in the cells treated with Lipofectamine/DNA, L6/DNA, and L5a/DNA complexes in the absence and presence of 113 mM calcium chloride. Significant increases of gene expression intensity and transfection efficiency were observed in treatment groups exposed to Lipofectamine/DNA, L6/DNA, and L5a/DNA complexes in the absence and presence of calcium chloride (Fig 6B and 6C). Further, calcium chloride significantly increased the apparent extent of gene expression in the cells treated with L6/DNA and L5a/DNA complexes, while decreasing gene expression in the cells treated with Lipofectamine/DNA complexes (Fig 6B). However, calcium chloride did not affect transfection efficiency in groups treated with Lipofectamine/DNA, L6/DNA, or L5a/DNA complexes (Fig 6C). These results indicate that the treatment with calcium chloride enhances CPP-mediated gene expression levels, without affecting CPP-mediated transfection efficiency.

### Correlations between electrostatic interactions and transfection ability of CPPs

**Fig 7:**
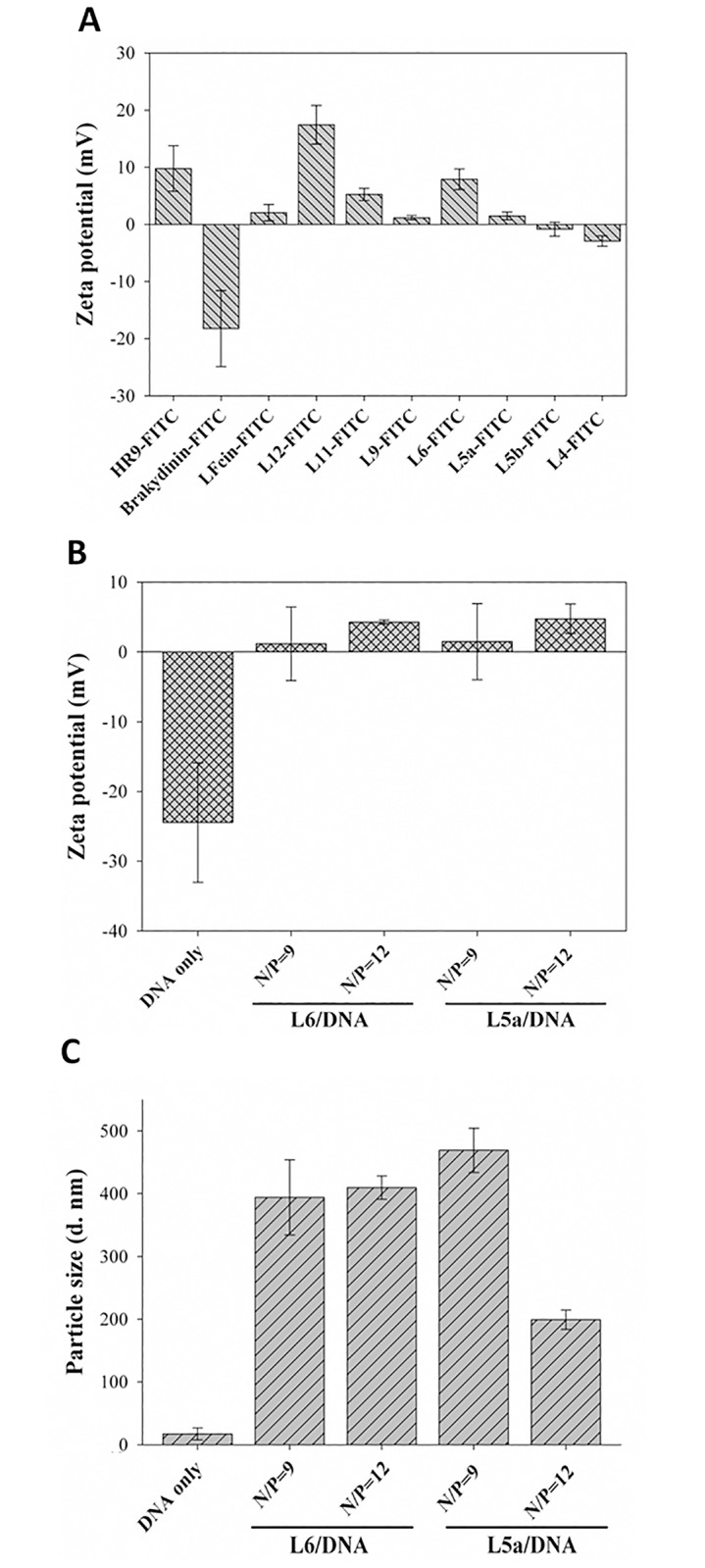
Zeta-potential and particle size analyses of CPPs, DNA, and CPP/DNA complexes. (A) Zeta-potentials of CPPs. HR9-FITC, bradykinin-FITC and eight FITC-labeled LFcin derivatives were dissolved in double deionized water and measured using a Zetasizer Nano ZS. Data are presented as mean ± SD from five independent experiments in each treatment group. (B) Zeta-potentials of DNA and CPP/DNA complexes. L6 and L5a were premixed with DNA at N/P ratios of 9 and 12. The DNA and CPP/DNA complexes were then dissolved in double deionized water and measured using a Zetasizer. Data are presented as mean ± SD from five independent experiments in each treatment group. (C) Particle sizes of DNA and CPP/DNA complexes. L6 and L5a were premixed with DNA at N/P ratios of 9 and 12. The DNA and CPP/DNA complexes were then dissolved in double deionized water and measured using a Zetasizer. Data are presented as mean ± SD from five independent experiments in each treatment group.

To evaluate the correlations between positive charge and protein transduction ability of CPPs, zeta-potential analyses were performed. FITC-labeled LFcins of various lengths were dissolved in double deionized water, and zeta-potentials were measured using a Zetasizer. Bradykinin-FITC, L5b-FITC, and L4-FITC were negatively charged (Fig 7A), while HR9-FITC and the other FITC-labeled LFcins exhibited positive potentials. L5a-FITC and L5b-FITC contain the same penta-residue compositions, but vary in sequence. It should be noted that zeta value of L5a-FITC was 1.5 ± 0.7 mV, while that of L5b-FITC was –0.8 ± 1.2 mV. This probably accounts for the differences of protein transduction ability between L5a-FITC and L5b-FITC. The surface charge of plasmid DNA contained a zeta-potential of –24.4 ± 8.5 mV (Fig 7B). Zeta values of L6/DNA complexes were 1.2 ± 5.3 mV and 4.3 ± 0.3 mV at N/P ratios of 9 and 12, respectively; while the potentials of L5a/DNA complexes were 1.5 ± 5.4 mV and 4.8 ± 2.1 mV at N/P ratios of 9 and 12, respectively. The size of DNA particle was 17.2 ± 9.7 nm in diameter (Fig 7C). Particle sizes of L6/DNA complexes were 394.1 ± 59.8 mm and 409.7 ± 18.5 nm at N/P ratios of 9 and 12, respectively. The sizes of L5a/DNA complexes were 469.1 ± 35.4 nm and 199.2 ± 15.4 nm at N/P ratios of 9 and 12, respectively. These data correspond to the results in the gel retardation assay (Fig 3) and transfection efficiency (Fig 5C) of CPP/DNA complexes. These results agree with our previous report that electrostatic interactions of CPP/cargo complexes predicts transfection efficiency [44].

### Cytotoxicity of CPP-mediated DNA delivery

**Fig 8:**
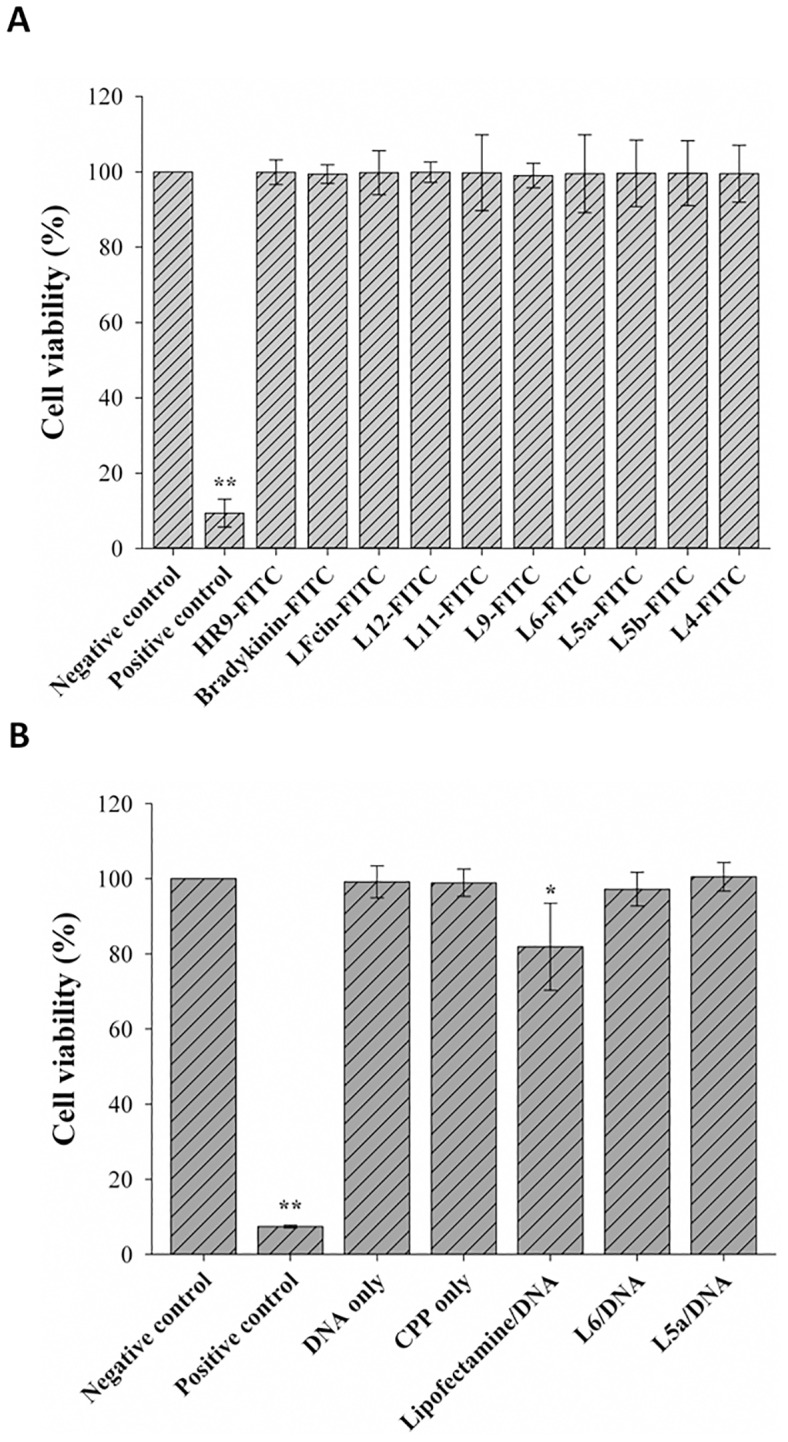
Cytotoxicity of CPPs, DNA, and CPP/DNA complexes. (A) Cytotoxicity of CPPs. Cells were treated with 10 μM HR9-FITC, bradykinin-FITC, or eight FITC-labeled LFcin derivatives at 37°C for 24 h. Cells treated with serum-free medium and 100% DMSO at 37°C for 24 h served as negative and positive controls, respectively. The MTT assay was used to evaluate cytotoxicity. Significant differences from the negative control at *P*<0.01 (**) are indicated. Data are presented as mean ± SD from five independent experiments in each treatment group. (B) Cytotoxicity of DNA and CPP/DNA complexes. Cells were treated with 1.6 μg DNA, 10 μM CPPs, Lipofectamine 2000/DNA, or CPP/DNA complexes prepared at an N/P ratio of 12 at 37°C for 24 h. Significant differences from the negative control at *P*<0.01 (**) are indicated. Data are presented as mean ± SD from five independent experiments in each treatment group.

To understand the cytotoxicity of LFcins of various lengths and the CPP/DNA complexes, the MTT assay was conducted. Cells were treated with 10 μM of FITC-labeled LFcins for 24 h, and then analyzed using the MTT assay. Cytotoxicity was not detected in cells treated with any FITC-labeled CPPs or nonCPP (Fig 8A). To evaluate the toxicity of CPP/DNA complexes, cells were treated with DNA, CPP alone, Lipofectamine/DNA, L6/DNA, or L5a/DNA complexes. Cell viabilities were close to 100%, expect for cells exposed to Lipofectamine/DNA complexes (Fig 8B). Mild cytotoxicity was shown in the cells transfected with Lipofectamine/DNA complexes. These results demonstrate that LFcins will be a safe vehicle to carry DNA cargos into cells.

## Discussion

In this study, we demonstrate that L5a can efficiently deliver noncovalently complexed plasmid DNA into cells. The *EGFP* reporter gene assay confirmed that plasmid DNA delivered by L5a can be actively expressed by cells at both the mRNA and protein levels. Zeta-potentials of CPP/DNA complexes correlated with transfection efficiency [44], emphasizing the importance of electrostatic interactions of CPP/DNA complexes with plasma membranes. Cell viability assay confirmed that none of the components of the LFcin CPPs and CPP/DNA complexes are cytotoxic. It shall be noted that both L6 and L5a can efficiently deliver plasmid DNA into cells as efficiently as the commercial Lipofectamine 2000, but with much less cytotoxicity.

L5a characterized in the current study represents one of the shortest CPPs discovered, with only five amino-acid residues (RRWQW). An anti-inflammatory peptide-6 (AIP6) containing five amino acids (RLRWR) has been reported as a CPP to transduce cell *in vitro* and *in vivo* [31]. This AIP6 has been claimed to be the shortest CPP [30]. CPP-mediated delivery of siRNA into HeLa cells with high transfection efficiency without immunogenicity or cytotoxicity was demonstrated with bLFcin_6_ (RRWQWR, corresponding to L6 in the present study) [30]. Our present results with L6 in human cells are consistent with these earlier results of bLFcin_6_. The structure of bovine LFcin revealed by nuclear magnetic resonance is a distorted antiparallel β-sheet, which could adopt either a helical or sheetlike conformation to interact with cellular membranes [45]. A disulfide bond is essential to stabilize the β-sheet structure of LFcin [27]. This shorter bLFcin_6_ with cell-penetrating properties provides the active center of the antimicrobial activity [24,30]. On the other hand, the CPP_5_ (RWQWR, corresponding to L5b in the present study) has less internalization activity [30]. Our present results with L5b agree with these studies of CPP_5_, and L5b appears to be a nonCPP. Both L5a and L5b contain the same five amino acid residues, but in different sequences. This slight difference of only one amino acid in sequence has significant functional consequences: L5a possesses intrinsic and potent cell-penetrating properties, while L5b and L4 (RWQW) do not (Figs 1, 2 and 7).

CPPs have been used in a wide number of applications, ranging from simple cell culture transfection to the systemic delivery of therapeutics. One of the first biomedical applications of CPPs was delivery of nucleic acids into cells [46]. Plasmid DNA noncovalently complexes with CPPs through electrostatic attraction, and the resulting complexes are delivered into cells [47]. Additionally, condensation of nascent CPP/DNA complexes by calcium chloride produces small (100–140 nm) and stable nanoparticles leads to the enhancement of gene expression levels [48]. Our present results showed that the treatment of calcium chloride enhances CPP-mediated gene expression levels (Fig 5). Collectively, these data are in agreement with our previous results that CPPs are able to efficiently deliver DNA into live cells or organisms [49]. We found that the treatment of calcium chloride does not affect the CPP-mediated transfection efficiency, but increase the gene expression levels (Fig 6). One possible reason is that calcium chloride condenses nascent plasmid DNA to form stable and small nanoparticles with 100–140 nm in diameter, leading to the enhancement of gene expression [48]. Another possible reason is that calcium ion may activate transcription factors to facilitate gene expression through its downstream signaling effectors; for instance, calcium/calmodulin-dependent protein kinase II (CaMKII), protein kinase C (PKC), and cAMP response element-binding protein (CREB) [50].

Macromolecular therapeutics involving intracellular delivery of enzymes, oligonucleotides, siRNAs, proteins/peptides, and large synthetic molecules, can potentially be used to treat human diseases by targeting specific intracellular molecular pathways, modulating biological responses, and potentiating intracellular effects [51]. However, the major drawback of macromolecules is their limited bioavailability. While multiple classes of delivery carriers have been developed, safe and robust uptake formulations are still being developed. CPPs can be potential targeting ligands for cancer therapeutics or cellular delivery systems [46,51–53]. Moreover, CPPs have been demonstrated to selectively cross the blood-brain barrier *in vivo* in mice [54]. Recently, clinical data involving CPP delivery of macromolecules have emerged from over 25 completed Phase I and Phase II clinical trials evaluating the safety and efficacy of CPPs *in vivo* [51], and there is currently one ongoing Phase III clinical trial utilizing a CPP-mediated delivery protocol [55]. Accordingly, combining CPPs with macromolecular drug designs is on the edge of equipping us with effective delivery vectors to treat cancers, viral infections, and other diseases [51].

## Conclusions

Bovine lactoferricin L5a (RRWQW) is capable of forming stable complexes with plasmid DNA at the optimized N/P ratio of 12 and deliver DNA into human A549 cells. L5a could deliver plasmid DNA encoding the *EGFP* reporter gene that was expressed by the cells. LFcin CPPs and CPP/DNA complexes were not cytotoxic. Thus, this novel L5a is one of the shortest functional CPPs characterized to date, and could provide an efficient and safe tool for cellular gene delivery.

## Supporting Information

S1 Fig Cellular internalization of FITC-labeled LFcins by dosage-dependent analysis. FITC-labeled LFcin derivatives at 0, 1, 5, 10, and 30 μM concentrations were incubated with human A549 cells for 1 h before measuring cellular uptake using a flow cytometer. Cells without any treatment served as internal controls. Cells were treated with HR9-FITC and bradykinin-FITC as positive and negative controls, respectively.
(TIF)

S2 Fig Cellular internalization of FITC-labeled LFcins by time-course analysis. Cells were treated with FITC-labeled LFcins of eight different lengths for 0, 5, 10, 20, 30 and 60 min. Cellular internalization ability was then analyzed using a flow cytometer.
(TIF)

## Abbreviations

AIP6: anti-inflammatory peptide-6
BFP: blue fluorescent protein
CMV: cytomegalovirus
CPP: cell-penetrating peptide
EGFP: enhanced green fluorescent protein
FITC: fluorescein isothiocyanate
GFP: green fluorescent protein
HR9: histidine-rich nona-arginine
LF: lactoferrin
LFcin: lactoferricin
MTT: 1-(4,5-dimethylthiazol-2-yl)-3,5-diphenylformazan
N/P: nitrogen (NH_3_^+^)/phosphate (PO_4_^−^)
PBS: phosphate buffered saline
PTD: protein transduction domain
RFP: red fluorescent protein
Tat: transactivator of transcription
